# A computational roadmap to electronic drugs

**DOI:** 10.3389/fnbot.2022.983072

**Published:** 2022-10-31

**Authors:** Andreas Rowald, Oliver Amft

**Affiliations:** ^1^ProModell Group, Chair of Digital Health, Department of Medical Informatics, Biometry and Epidemiology, Friedrich-Alexander University Erlangen-Nuremberg, Erlangen, Germany; ^2^Intelligent Embedded Systems Lab, Institute of Computer Science, University of Freiburg, Freiburg im Breisgau, Germany; ^3^Hahn-Schickard, Freiburg, Germany

**Keywords:** computational modeling, digital twins, neurostimulation, neuromodulation, neurological disorders, psychiatric disorders

## Abstract

A growing number of complex neurostimulation strategies promise symptom relief and functional recovery for several neurological, psychiatric, and even multi-organ disorders. Although pharmacological interventions are currently the mainstay of treatment, neurostimulation offers a potentially effective and safe alternative, capable of providing rapid adjustment to short-term variation and long-term decline of physiological functions. However, rapid advances made by clinical studies have often preceded the fundamental understanding of mechanisms underlying the interactions between stimulation and the nervous system. In turn, therapy design and verification are largely driven by clinical-empirical evidence. Even with titanic efforts and budgets, it is infeasible to comprehensively explore the multi-dimensional optimization space of neurostimulation through empirical research alone, especially since anatomical structures and thus outcomes vary dramatically between patients. Instead, we believe that the future of neurostimulation strongly depends on personalizable computational tools, i.e. Digital Neuro Twins (DNTs) to efficiently identify effective and safe stimulation parameters. DNTs have the potential to accelerate scientific discovery and hypothesis-driven engineering, and aid as a critical regulatory and clinical decision support tool. We outline here how DNTs will pave the way toward effective, cost-, time-, and risk-limited electronic drugs with a broad application bandwidth.

## Introduction

Neurostimulation strategies can be represented in a three-layer structure, where (i) electric fields are applied at selected body locations to (ii) modulate activity of certain nervous structures, i.e. neural targets, which in turn interact with the architecture of the nervous system to (iii) produce a physiological outcome. In much of contemporary neurostimulation, we understand little of each layer, or how the outcomes of each layer transitions to the next layer. In the absence of clear mechanistic understanding critically important stimulation parameters for neurostimulation therapies are based solely on previous empirical evidence, and on the short-term stimulation effects in terms of the patient's clinical improvement (Sun and Morrell, 2014). The largely clinically-empirical therapy design, carries with it several problems including issues relating to time- and cost-effectiveness, safety related concerns, and complications during clinical implementation.

### Neurostimulation research is slow and cost-intensive

Scientific advancements in neurostimulation are intertwined with months or years of therapy optimization in multidisciplinary expert teams to find a narrow optimum of stimulation parameters (Capogrosso and Lempka, 2020). To our knowledge, a comprehensive set of all parameters that influence the therapeutic outcome of neurostimulation have not been identified yet, but parameters certainly include electrode location, stimulation amplitude, frequency, pulse shape, timing, and physiological parameters of the nervous system. As mechanisms of action are not yet fully understood, therapy design is a Sisyphean task. Parameters differ between individuals due to anatomical differences, and even on a person-specific level, the optimal configuration may be changing due to short-term fluctuations and long-term decline (McIntyre and Foutz, 2013; Capogrosso and Lempka, 2020; Lempka et al., 2020; Rowald et al., 2022). Therapy verification in clinical trials is constrained by significant costs and strict, but necessary, regulatory constraints. It is simply infeasible to comprehensively explore and optimize the multi-dimensional design space of neurostimulation through clinical research alone.

### Therapy design does not fully address safety concerns

Rapid research advancements often address safety concerns by reporting adverse events, without further analysis of the origin of potential side effects. Neurostimulation strategies should optimally apply electric current in a body region that limits health and comfort risks while being in direct contact with the neural target. In practice, neural targets are often hidden in deep and sensitive anatomical layers. Electric current must therefore often traverse several tissue layers, including neural substrates controlling diverse neurological functions (Hofstoetter et al., 2018). In turn, unwanted tissue layers are modulated by artificial stimuli and can yield unintended side effects. Safety concerns are further amplified by requirements for other medical implants, e.g. fixations, which can themselves manipulate electric fields, potentially leading to local amplitude peaks or heat deep tissue layers. Similarly, interactions between neurostimulation devices and external electromagnetic fields raise several safety concerns and may disrupt patient-related outcomes. Particularly MRI-safety is a concern as regular medical imaging assessments are advisable in patients treated with neurostimulation devices to address several systematic issues such as electrode migration, yet electromagnetic fields present in MRI scanners may lead to implant movements, tissue heating, or electromagnetic interference (Nazarian et al., 2013). Safety claims of neurostimulation devices can fail to address interpatient variabilities in usage, raising concerns that interventions will cause more harm than good (McCall et al., 2019).

### Neurostimulation does not translate from research frontier to regular care

Regardless of all obstacles, some neurostimulation strategies have become clinically approved, including Deep Brain Stimulation (DBS) for movement disorders and Spinal Cord Stimulation (SCS) for neuropathic pain. Simultaneously, obstacles faced in therapy design have seamlessly been transferred to regular care. Clinicians are tasked to perform stimulation optimization to identify appropriate parameters for every patient, in addition to their regular clinical duties, and without extensive knowledge of electric field distributions and interactions thereof with neural structures. In turn, clinicians are asking for patient-stratification strategies, as in their absence clinically-available neurostimulation strategies have been rendered treatments of last resort (Simpson, 2006). Yet, the efficacy of neurostimulation strategies changes on a timescale not consistently warranting specialist intervention, as interactions between fixed electrodes and neural tissues can be influenced by rapidly fluctuating parameters such as changes in body position (Ross and Abejón, 2014). In the absence of self-regulating neurostimulation systems, patients employ manual regulating strategies, potentially resulting in suboptimal therapy delivery (Ross and Abejón, 2014).

## Computational modeling for neurostimulation

In parallel to clinical-empirical research, computational modeling has a rich history in neurostimulation. Computational models aim to reduce the complexity of neurostimulation by providing abstract and simplified representations of neurostimulation strategies in the absence of noisy confounding factors. In the 1980s, a combination of Finite Element Models (FEMs) and circuit models of nerve axons were used to predict sensory thresholds of dorsal column and dorsal root afferent fiber pathways during SCS for pain control (Coburn, 1985; Coburn and Sin, 1985). The same methodology continues to be applied today for a variety of applications, including SCS for motor control, DBS for movement disorders, and peripheral nerve stimulation (Rattay et al., 2000; McIntyre and Foutz, 2013; Musselman et al., 2021). A new direction has emerged in recent years, whereby computational models account for interpatient variability. Patient-specific computational models have shown success in therapy design and clinical-decision making in various neurostimulation strategies, including DBS for movement disorders and SCS for motor control (Frankemolle et al., 2010; Lempka et al., 2020; Rowald et al., 2022). While yet rarely termed digital twins in the neurostimulation literature, patient-specific computational models attempt to represent relevant neurological properties and processes in a digital format that mirrors their physical counterparts in real-time, just as the term originated in manufacturing suggests.

**Figure 1:**
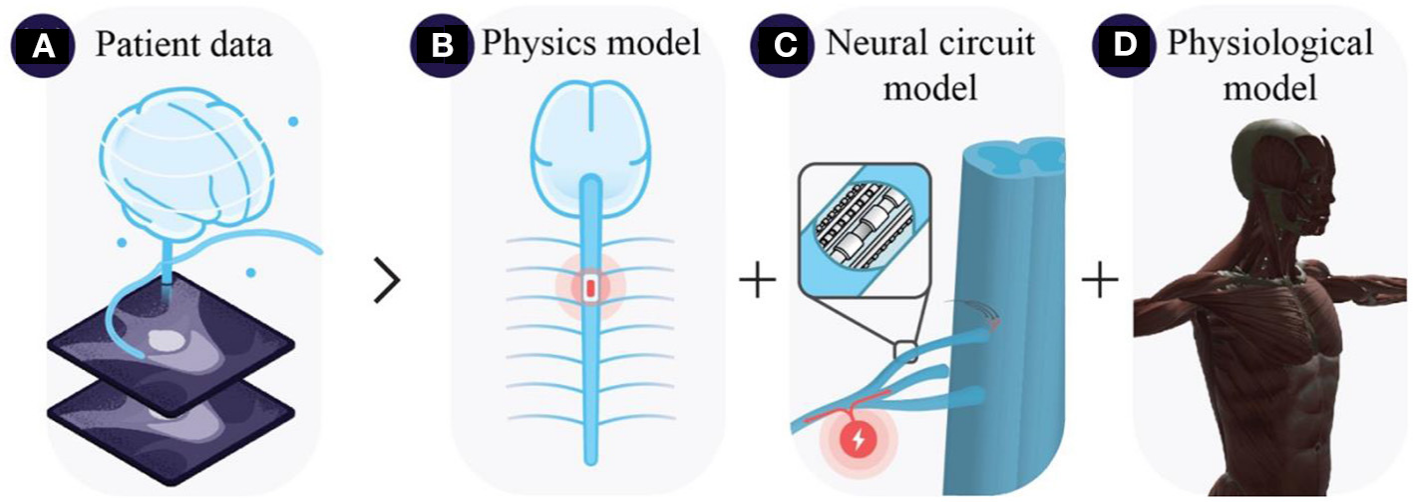
Three-layer neurostimulation modeling architecture, illustrating **(A)** extraction of patient data from monitoring systems e.g., MRI scans, **(B)** approximation of electric field distributions coupled with **(C)** neural circuit models, and **(D)** physiological end-organ models e.g., musculoskeletal models.

Digital twins in neurostimulation—i.e. DNTs—leverage patient data including medical imaging datasets to replicate the three-layer structure of neurostimulation strategies (Figure 1A). DNTs employ multi-physics frameworks to calculate the electric field distributions in the patient's anatomy (Figure 1B). Coupled with neural circuit models DNTs approximate interactions between stimulation and neural structures (Figure 1C). Approximated neural dynamics can be projected to computational approximations of end-organs to anticipate physiological outcomes (Figure 1D). DNTs are still in their infancy with many approaches approximating only parts of the multi-faceted system of neurostimulation strategies in a rudimentary fashion. However, we believe that the future of neurostimulation will strongly depend on the development of novel DNTs. DNTs have the capacity to accelerate the identification of neural targets to selectively modulate physiological functions (see Section Computational guidance for hypothesis-driven neurostimulation). DNT-guidance opens the possibility to identify effective, safe, and robust stimulation parameters efficiently and continuously for the selective modulation of neural targets—and thus physiological outcomes—on a per subject basis (see Section Readying the electronic drug market with digital neuro twins). If DNTs of the future overcome certain challenges (see Section Open challenges in digital neuro twin modeling), we believe they will transition neurostimulation strategies to electronic drugs (e-drugs), that deliver specific treatments in an adjustable and directed manner to targeted tissues and organs, thus alleviating limitations regarding spatial and temporal aspects of treating diseases (Vadlapatla et al., 2017).

## Computational guidance for hypothesis-driven neurostimulation

DNTs are natural candidates to dissect interactions between neurostimulation strategies and the nervous system and may even be useful in elucidating how these interactions relate to physiological outcomes.

### DNTs deal with patient-specific variability

Although it remains unclear how many parameters influence neurostimulation outcome, interpatient variability is one important parameter. With ever-increasing medical imaging capacities and sophisticated computer vision technologies, interpatient variability will be an accountable parameter and thus compensated by a model-guided neurostimulation strategy. Already today, there are several examples of medical imaging capacities having been incorporated to generate DNTs accounting for patient-anatomy and even functional neural connectivity (Frankemolle et al., 2010; Lempka et al., 2020; Rowald et al., 2022).

### Simulating neurostimulation physics reduces uncertainty

We believe that DNTs will allow us to put evidence relating to outcomes in perspective to hypothetical mechanisms of action. Patient-specific FEMs enable the approximation of electric fields in a patient's anatomy (Frankemolle et al., 2010; Lempka et al., 2020; Rowald et al., 2022). Replicating stimulation parameters from clinical observations in patient-specific FEMs reduces uncertainty in neurostimulation research by reducing the problem from a coupled physical-physiological problem to a predominantly physiological problem.

### DNTs initiate a paradigm shift in neurostimulation research

Patient-specific FEMs may be coupled with electrophysiological representations of neurons to provide a first-order approximation of the interactions between electric fields and the nervous system. In direct comparison with experimental outcomes, these anatomy-controlled approximations of neurostimulation patterns reveal which neural substrates, i.e., neural targets are activated, inhibited, or otherwise modulated in the presence of artificially applied electric fields. DNTs may then be employed in clinical studies to identify stimulation parameters that will selectively elicit the assumed mechanisms on a per-subject basis. Thus, computationally-guided clinical research may reveal which neural targets must be modulated in what manner to improve patient-related outcomes.

However, it remains unclear how the identified interaction between neural target and neurostimulation translates into physiological outcome and what the long-term patient-related consequences of this interaction may be. We believe these issues may be addressable by supplementing DNTs with artificial representations of hypothesized network architectures such as artificial neural networks and physical representations of physiological organs such as biomechanical models of the musculoskeletal system. DNTs that include neural network architectures and biomechanical models have already shown promise in approximating circuit-level activities of SCS for the recovery of locomotion (Formento et al., 2018).

## Readying the electronic drug market with digital neuro twins

Once neural targets have been identified, we believe that DNT will play a pivotal role in the efficient identification of effective, safe, and robust stimulation parameters.

### DNTs pave the way toward effective and safe e-drugs

Scientific and industrial bodies can leverage DNTs to efficiently search stimulation parameter spaces in large and diversified patient cohorts for Pareto-optimal solutions to maximize the selective recruitment of neural targets in the absence of confounding effects, such as thermal heating or the modulation of unwanted neural structures, thus accelerating the design and verification of neurostimulation e-drugs. Regulatory bodies are moving toward ensuring the efficacy, safety, and robustness of e-drugs in a virtual environment, warranting the swift translation of e-drugs to regular care (Morrison et al., 2018). Clinicians will benefit from personalized treatment planning tools based on DNTs, enabling the interactive design of subject-specific therapies.

### Rapid adjustments to short-term fluctuations and long-term decline

A major problem of classical, non-electronic pharmacological interventions is that they usually cannot rapidly adjust to short-term fluctuations of physiological function. Furthermore, resistance to medication may arise over time. Although current clinically-approved neurostimulation strategies show promise to maintain long-term improvements in quality of life, revision rates also remain high and technologies for rapid adjustments to short-term fluctuations have not yet reached the desired technology-ready status (Rolston et al., 2016; Edwards et al., 2017). Closed-loop neurostimulation is being actively investigated to provide solutions for rapid adjustment of patient-related outcomes. We believe that DNTs will play a key role in the development of closed-loop neurostimulation strategies as adjustments of stimulation parameters for effective and safe neurostimulation may be highly non-linear. For each neurostimulation strategy several factors influence the patient-related outcomes. Simple adjustments of stimulation parameters in the absence of DNT-guidance may result in confounding effects such as co-activation of unwanted tissues. Thus, we believe that e-drugs of the future will incorporate DNTs as a software component to calculate what stimulation is needed in a moment of time to either compensate for missing functionality or stimulate beneficial reorganization over time.

### E-drugs with broad application bandwidth

Neurostimulation strategies inherently have a broad application bandwidth, with technologies including DBS being clinically approved treatments for one condition i.e., movement disorders while simultaneously being actively investigated for a host of other disorders such as mood, memory, or sleep deficits. Particularly the location of electrodes plays an important role in which treatment modality a neurostimulation strategy may be used, indicating that different neural targets must be modulated. Recent advancements have demonstrated that advanced concepts of classical field theory including temporal interference can be used to achieve field focality in deep and hidden tissues of the nervous system without the electrodes being in direct contact with those tissues, thus paving the way toward ubiquitous e-drugs with a broad application bandwidth (Grossman et al., 2017). The hardware component of e-drugs will have the capacity to apply electric current in a region of the human body that is cost-, time-, and risk-limited and yet modulate a broad range of neural targets with different stimulation parameters. The efficient identification of stimulation parameters for different treatment modalities will be dictated by DNTs incorporated into the software components. Ongoing trends within the scientific and industrial community investigating the use of digital twins to efficiently identify personalized stimulation parameters will manifest DNTs in the support of several neurostimulation strategies, including non-invasive brain stimulation (Sanchez-Todo et al., 2018; Amunts et al., 2022).

## Open challenges in digital neuro twin modeling

DNTs must be rapidly generatable with limited expert supervision to ensure large-scale uptake in neurostimulation research, therapy design, verification, and clinical decision-making. Imaging sequences must be standardized across vendors and scanners that enable the accurate recapitulation of relevant tissues. Dedicated and automated computational pipelines must be developed to generate 3D models from imaging datasets, assign physical properties, discretize the geometry, perform multi-physics simulations, neurofunctionalize and translate the neural activity into physiological outcome, and incorporate sensor data. Optimization routines anticipating physiological outcomes must be tuned to clinical-empirical outcomes and tailored to patient-related needs. Although countless challenges associated with those steps can be imagined, we would like to highlight key issues that likely enable a breakthrough of DNTs.

### Limited understanding of mechanisms of action

The most important challenge of DNTs is that every theoretical model is only as good as the understanding of the underlying system. Unfortunately, much of the inner workings of the nervous system, its interactions with neurostimulation strategies, and how neural dynamics translate to physiological outcomes remain enigmatic. It is easy to argue, that due to our limited understanding of neuroscience and neurostimulation, all computational models thereof are useless. Contrarily, when attempting to replicate neurostimulation *in-silico*, we often understand what we do not yet understand, which is a critical information capable of driving experimental research (Capogrosso and Lempka, 2020). In turn, experimental observations will inevitably challenge the theoretical models and thus the cycle of scientific discovery repeats. It remains unclear, if DNTs will ever perfectly describe reality. Yet, it is important to remember the famous aphorism of George Box: “All models are wrong, but some are useful.” The primary challenge in computational neurostimulation will be to identify how to construct computational models that are useful for different purposes, be it scientific advancement, therapy design, or personalized treatment planning.

### Scientific-technical challenges of DNTs

It remains unclear what level of personalization and accuracy is sufficient to improve patient-related outcomes. It may be necessary to incorporate personalization steps for parameters such as tissue conductances and neural connectivities in the workflow using technologies including novel imaging modalities (Landelle et al., 2021; Rimpiläinen et al., 2021; Rocha et al., 2022). Further, there will be technical challenges in ensuring robust automation of the DNT workflows in the face of variabilities in patient data, user needs, computational proficiency, or available resources. In recent years, technological frameworks have started to emerge that provide semi-automated workflows for DNTs (Neufeld et al., 2013; Amunts et al., 2022). Some have been moved to cloud-based platforms, enabling users to outsource computational resources, while benefitting from intuitive and interactive workflows (Neufeld et al., 2018). DNT workflows may also need to be locally deployable on implantable or wearable devices to rapidly calculate effective and safe stimulation parameters during closed-loop neurostimulation. Computational resources will be a limiting factor and thus less computationally demanding, yet scientifically accurate modeling modalities must be developed.

### Socio-economic challenges of DNTs

There are several challenges to ensure ethical management of patient data and adherence to related data management principles and regulations. Similarly, technical challenges in the automation of DNT workflows may necessitate the usage of AI for purposes including image segmentation, thus opening the discussion of explainable AI for clinical-decision making. After all, computationally-guided neurostimulation will significantly impact the patient pathway, offering an additional, potentially more effective and safe treatment option to patients. This departure from the current patient pathway will have to be explained and justified to patients, clinicians, health system administrators, and policy makers. That last group is particularly important, as they will ultimate decide on the funding and resourcing of DNT-based services. Financial decision making will be crucial as many patients will depend on health insurances to pay for e-drugs. Given that today, the average direct costs of neurostimulation strategies tend to be larger than conventional medical interventions, there must be strong effectiveness, efficiency, and safety considerations to convince health insurances to pay for e-drugs (Mekhail et al., 2004; Dams et al., 2016; Niyomsri et al., 2020). For example, policy makers may subsidize effective and safe e-drugs to reduce the societal and economic burden of neurological and psychiatric disorders, many of which cause long-term disability (Deuschl et al., 2020).

## Discussion

Today's development state of neurostimulation may be considered analogous to that of cardiac implantable electronic devices of the 1950s−1960s. A mechanistic understanding of the heart's electric architecture and its interaction with external stimuli of the early cardiac implants resulted in contemporary cardiac pacing therapies being clinically approved, safe, efficient, and robust treatment for heart disorders. The innovation was feasible because researchers learned to understand the heart and leveraged their insight for therapy optimization. Unfortunately, we still understand much less about the nervous system today than about the heart. Yet, recreating what we do understand about the nervous system in computers not only gives us a first-order approximation of neurostimulation mechanisms but will also immediately reveal what we yet have to work on. We believe that DNTs can accelerate and streamline therapy conception, verification, and deployment beyond neurostimulation toward e-drugs. Computationally-guided and personalized e-drugs of the future will be effective and cost-, time-, and risk-limited interventions with a broad application bandwidth that (re-)establish functional capacity. What is needed now is the definition of computational workflows that can efficiently identify mechanisms of action and transfer this understanding into effective, safe, and robust stimulation parameters for neurostimulation strategies.

## Data availability statement

The original contributions presented in the study are included in the article/supplementary material, further inquiries can be directed to the corresponding author.

## Author contributions

AR and OA conceived and wrote the presented manuscript. Both authors contributed to manuscript revision, read, and approved the submitted version.

## Funding

This work was funded by the Bundesministerium für Bildung und Forschung BMBF#01ZZ2016.

## Conflict of interest

The authors declare that the research was conducted in the absence of any commercial or financial relationships that could be construed as a potential conflict of interest.

## Publisher's note

All claims expressed in this article are solely those of the authors and do not necessarily represent those of their affiliated organizations, or those of the publisher, the editors and the reviewers. Any product that may be evaluated in this article, or claim that may be made by its manufacturer, is not guaranteed or endorsed by the publisher.
